# SEXUALLY TRANSMITTED INFECTION KNOWLEDGE AMONG OLDER ADULTS: PSYCHOMETRICS AND TEST-RETEST RELIABILITY

**DOI:** 10.1093/geroni/igz038.1105

**Published:** 2019-11-08

**Authors:** Matthew L Smith, Tammy Coffey, Caroline D Bergeron, Enbal Shacham, Heather H Goltz, Ali Boolani

**Affiliations:** 1 Center for Population Health and Aging, Texas A&M University, College Station, Texas, United States; 2 Clarkson University, Potsdam, New York, United States; 3 Bexar County Community Health Collaborative, San Antonio, Texas, United States; 4 Saint Louis University, St. Louis, Missouri, United States

## Abstract

Sexually transmitted infections (STI) among older adults have dramatically increased in recent years, especially among those who are widowed and divorced. The purposes of this study were to: (1) identify STI-related knowledge among older adults; (2) report the psychometric properties of a tool commonly used to assess STI-related knowledge among youth and young adults; and (3) determine the test-retest reliability of the tool. Data were analyzed from 43 adults between the ages of 65 and 94 using a 27-item Sexually Transmitted Disease Knowledge Questionnaire (STD-KQ). Participants completed identical instruments on two separate days with approximately two weeks between. After responses were coded for correctness, composite scores were created. Cronbach’s reliability coefficients were calculated to determine response consistency, and Pearson’s r coefficients were used to assess test-retest reliability. Of 27 possible correct answers, participants reported an average of 11.6 (±6.6) correct responses on Day 1 and 11.7 (±7.3) correct responses on Day 2. Cronbach’s alpha coefficients for the 27-item composite scale were high for both days (0.89 and 0.92, respectively), which indicates strong response consistency. Pearson’s r coefficients were high between responses for the 27-item composite scale on Days 1 and 2 (r=0.88, P<0.01), which indicates strong test-retest reliability. Pearson’s r coefficients were high between responses for all but three of the 27 items when assessed separately. Findings suggest the utility of the STD-KQ to assess STI knowledge among older adults. However, the consistently low knowledge scores highlight the need for educational interventions among this population.

